# Partial Cystectomy after Neoadjuvant Chemotherapy: Memorial Sloan Kettering Cancer Center Contemporary Experience

**DOI:** 10.1155/2014/702653

**Published:** 2014-11-13

**Authors:** Wassim M. Bazzi, Ryan P. Kopp, Timothy F. Donahue, Melanie Bernstein, Paul Russo, Bernard H. Bochner, Sherri M. Donat, Guido Dalbagni, Harry W. Herr

**Affiliations:** The Urology Service, Department of Surgery, Memorial Sloan Kettering Cancer Center, 1275 York Avenue, New York, NY 10065, USA

## Abstract

*Objective*. To report our contemporary experience with partial cystectomy after neoadjuvant chemotherapy.* Patients and Methods*. Retrospective review of patients who underwent neoadjuvant chemotherapy and partial cystectomy for urothelial cell carcinoma of the bladder at Memorial Sloan Kettering Cancer Center from 1995 to 2013. Log-rank test and Cox regression models were used to analyze variables possibly associated with recurrence-free, advanced recurrence-free (free from recurrence beyond salvage with intravesical therapy or radical cystectomy), and overall survival.* Results*. All 36 patients had a solitary tumor <5 cm in size. Twenty-one patients (58%) achieved cT0 following neoadjuvant chemotherapy with 7 (33%) having residual disease at PC. At last follow-up, 19 (53%) patients had recurrence, 15 (42%) had advanced recurrence, 10 (28%) died of disease, and 22 (61%) maintained an intact bladder. Median follow-up of those who were with no evidence of disease was 17 months. On univariable analysis, after neoadjuvant chemotherapy positive nodes on imaging and positive surgical margin at partial cystectomy were both associated with worse recurrence-free, advanced recurrence-free, and overall survival. Five-year recurrence-free, advanced recurrence-free, and overall survival were 28%, 51%, and 63%, respectively.* Conclusion*. Partial cystectomy following neoadjuvant chemotherapy provides acceptable oncologic outcomes in highly selected patients with muscle-invasive bladder cancer.

## 1. Introduction

In 2014, it is estimated that there are 608,620 bladder cancer survivors living in the United States, and an additional 74,690 cases will be diagnosed [1]. Neoadjuvant chemotherapy (NC) followed by radical cystectomy (RC) is now considered the standard of care for muscle-invasive bladder cancer after numerous trials demonstrated a survival benefit, most notably in patients with advanced pathologic stage disease [2–4].

For highly selected patients, bladder-sparing surgery such as transurethral resection of bladder tumor (TURBT) [5, 6] or partial cystectomy (PC) [7–10] may provide similar oncologic outcomes to RC while maintaining bladder and sexual functions. Of the two bladder-sparing options, PC has advantages over TURBT as a third of patients are understaged with TURBT [11] and PC allows for full thickness examination of the bladder wall and concurrent lymphadenectomy resulting in more accurate staging and prognosis [7].

Previous studies have shown PC to provide comparable oncologic outcomes to RC after NC. Our institution previously reported on 111 patients who received neoadjuvant methotrexate-vinblastine-doxorubicin-cisplatinum (M-VAC) chemotherapy followed by TURBT. Of the 60 patients achieving cT0, 15 subsequently underwent PC and 17 underwent RC. Ten-year metastasis-free survival for the 15 patients who underwent PC was 73% with 53% of patients having their bladders intact, compared to the 65% 10-year metastasis-free survival for patients undergoing RC [11]. Similarly in a prospective trial conducted by Sternberg et al. [5], 104 patients with muscle-invasive bladder cancer underwent TURBT after 3 cycles of M-VAC chemotherapy and 49% of the cohort achieved cT0 after NC. Of these 104 patients, 13 patients with a solitary lesion underwent PC, while 39 patients underwent RC. For patients undergoing PC, 5-year survival was 69% with 4 patients alive after a median follow-up of 88 months (range 16–158) compared to 5-year survival of 46% with 15 patients alive after a median follow-up of 45 months (range 4–172) for patients undergoing RC.

We herein report our contemporary experience with a highly select cohort of patients who received neoadjuvant chemotherapy followed by PC performed for curative intent at a single tertiary institution.

## 2. Patients and Methods

### 2.1. Study Population

In this institutional review board-approved retrospective study, we identified patients who underwent PC at Memorial Sloan Kettering Cancer Center from 1995 to 2013 (*n* = 331). Only patients who underwent NC for urothelial cell carcinoma of the bladder followed by PC with curative intent were included in our study (*n* = 36). These were not consecutive cases and all patients underwent restaging TURBT at our institution prior to and after NC with cystoscopic mapping of bladder tumor/scar. Lymph nodes dissection data was available on 31 patients. All patients were followed up postoperatively with imaging and cystoscopy every 3 months for 2 years with widening of surveillance interval after.

### 2.2. Variables and Outcomes

We recorded data for clinical and pathologic variables including age, gender, race, tumor size and focality, histology, presence of carcinoma in situ (CIS), cross-sectional imaging, type and duration of NC, clinical and pathologic stages according to American Joint Committee on Cancer 2010 TNM staging 7th edition, surgical margin (SM), disease status, and cause of death. Survival outcomes included recurrence-free survival (RFS) which was defined as freedom from recurrence after PC, advanced recurrence-free survival (ARFS) which was defined as freedom from recurrence after PC beyond salvage with intravesical therapy or RC, and overall survival (OS).

### 2.3. Statistical Analysis

Kaplan-Meier survival estimates were generated with time measured from the date of PC to the date of event (recurrence, advanced recurrence, and death) or last follow-up. Using univariate Cox regression for continuous and log-rank text for categorical variables, we analyzed variables for association with RFS, ARFS, and OS. All probabilities were two-sided, and a *P* value <0.05 was considered significant for all analyses. All data were analyzed using STATA version 12.0 (StataCorp, College Station, TX, USA).

## 3. Results

A total of 36 patients underwent NC followed by PC (Table 1). Median age for the cohort was 70 years old (interquartile range (IQR) 58.8–76.8). All tumors were solitary, less than 5 cm in diameter, and 22 (61%) had a variant histology. Six tumors were located in the anterior wall, 19 in the lateral wall, 5 in the posterior wall, 3 in the base/trigone, and 3 in a diverticulum. Chemotherapy characteristics are described in Table 1; most patients received platinum-based chemotherapy with 20 patients (56%) having received gemcitabine and cisplatin combination. Unilateral ureteral reimplantation was performed in 7 patients at the time of PC to achieve SM in all cases. Margin status was evaluated by intraoperative frozen sections at PC in all patients.

As shown in Table 1, prior to NC, 22 (61%) patients had cT2 disease, 21 (58%) had cTis, and 6 (17%) had cN+. All patients were clinically restaged after NC (Tables 1 and 2) with 21 (58%) patients achieving cT0, 3 (8%) having cTis, and 4 (11%) having cN+. Of the 4 patients with cN+ after NC, 3 had cN+ before NC, and information prior to NC was unavailable for 1 patient.

As shown in Tables 1 and 3, PC pathologic findings were pT0 in 18 (50%) patients, pTis in 6 (17%), pN+ in 4 (11%), and SM+ in 3 (8%). The SM+ was perivesical in 1 patient with pT3 disease and pTis at margin in 2 patients with pT3 disease and pT2 disease. All 3 patients with SM+ experienced recurrence and died of disease (DOD) at 5, 10, and 43 months. Of the 21 patients who were cT0 after NC, 7 (33%) had residual bladder disease in the PC specimen (Table 3).

At last follow-up, 19 (53%) patients had recurrence, 15 (42%) had advanced recurrences, 10 (28%) died of disease, and 1 died of another cause. Twenty (56%) patients were with no evidence of disease (NED) after median follow-up of 17 months (IQR 9.4–38.2), with 15 having had no recurrences. Twenty two (61%) patients had an intact bladder. Four (11%) patients had NED after bladder cancer recurrences. Two of these 4 patients underwent intravesical bacillus Calmette-Guerin (BCG) treatment at 8 and 23 months after PC and the other 2 patients underwent RC at 23 and 43 months after PC. One patient had NED after irradiation of brain metastasis at 24 months after PC. Four were alive with disease (AWD): 2 patients having disease in the pelvis and 2 patients having disease in retroperitoneum.

Of the 19 (53%) patients who experienced recurrence (Table 4) 9 had recurrence in the bladder with 6 in the bladder only. Of the 9 patients with bladder cancer recurrences, 5 were at the resection site and 2 had SM+ at PC. Of the 6 patients with bladder-only recurrences, 2 patients had cTis, received BCG therapy, and have been NED to date; 2 patients had cT2 and cT1/cTis tumors, underwent RC, and remained disease free; 1 patient had cTis and received BCG but developed distant metastases (lung and adrenal) without further bladder recurrences 7 years after PC and eventually died of disease. Another patient developed persistent cT1/cTis disease that was managed with repeating TURBT and BCG before a failed attempt at RC and later died of disease.

**Figure 1:**
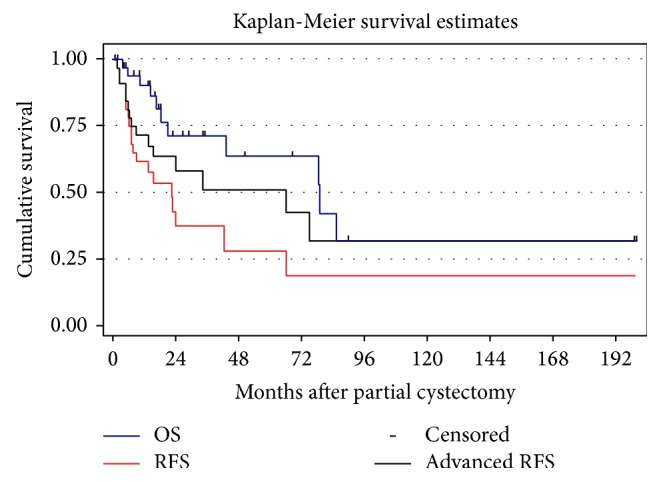
Recurrence-free survival, advanced recurrence-free survival, and overall survival for 36 patients who underwent a partial cystectomy.

As shown in Table 5, median time to recurrence was 23 months (IQR 5.9–66.2), median time to advanced recurrences was 66 months (7.2—not reached), and median time to death was 79 months (20.6—not reached). Kaplan-Meier survival estimates for 2- and 5-year RFS, ARFS, and OS were 37% and 28%, 58% and 51%, and 71% and 63%, respectively (Figure 1).

Results from univariable analyses are shown in Table 6. Before NC, clinical stage >cT2 was associated with both worse RFS (*P* = 0.03) and ARFS (*P* < 0.01). After NC, the presence of CIS was associated with worse OS (*P* = 0.04) and cN+ was associated with worse RFS (*P* < 0.01), ARFS (*P* < 0.01), and OS (*P* < 0.01). Following PC, presence of ≥pT2 disease was associated with worse RFS (*P* = 0.02) and ARFS (*P* = 0.01), pN+ was associated with worse RFS (*P* = 0.04), and SM+ on final pathology was associated with worse RFS (*P* = 0.01), ARFS (*P* = 0.04), and OS (*P* < 0.01). Multivariable statistical analysis was not performed for small number of events in our cohort.

## 4. Discussion

Our findings have been consistent with those of the literature with regard to oncologic outcomes in patients undergoing PC after NC. In our series, 74% of patients were downstaged after NC with 58% having a complete clinical response. Of the patients who achieved cT0 after NC, 7 (33%) had evidence of residual disease within the resected specimen at PC, which is close to 30% of understaging with TURBT alone reported by our institution's Herr and Scher study [12]. Two- and 5-year overall survival were 71% and 63%, respectively, which were comparable to oncologic outcomes of other studies involving PC after NC [5, 11, 12] and RC [13].

In our study, at last follow-up 19 patients (53%) had experienced recurrence and 24 (67%) were alive with 22 patients (61%) having retained an intact bladder. Nine patients had recurrence in the bladder with 5 at the suture line. In RC series by Stein et al. [13], local pelvic recurrence only occurred in 6%–13% of cases depending on radical cystectomy pathology and in our series 5 (14%) patients had recurrence in the pelvis with only 2 having isolated pelvic recurrences. Both of these patients with pelvic-only recurrences had negative surgical margins at PC with only 1 patient having recurrence on the ipsilateral side of the previous tumor.

For our study, advanced recurrence was defined as presence of disease that cannot be treated with salvage intravesical therapy or RC, which differs from previous reports of disease recurring in the bladder muscle and beyond [7, 8]. We believe that, with the improved quality of surveillance cross-sectional imaging and follow-up cystoscopies, disease control can still be achieved despite recurrences. This was evident in the 6 patients with isolated bladder recurrence as only 1 patient experienced disease progression and died of disease. We also noted that 53% of patients who experienced recurrence had no disease in bladder and hence it is unclear whether a RC would have altered their disease course.

In our previous report by Holzbeierlein et al. [7], the authors noted that presence of CIS preoperatively was associated with local recurrence and SM+ and pN+ were associated with advanced recurrence, as defined by muscle invasion and beyond. Though this series differs for lack of association of CIS with recurrence, we noted similar findings with SM+ and pN+. All 3 patients with SM+ on final pathology experienced recurrence and died of diseasewith SM+ being associated with worse RFS, ARFS, and OS on univariable analysis. Similarly, 4 patients had cN+ after NC with 2 eventually having pN+ at PC. Of those 2 patients with pN+, one was AWD at 7.3 months of follow-up and the other died of disease at 16.8 months of follow-up. cN+ after NC was associated with worse RFS, ARFS, and OS on univariable analysis.

Unlike in Kassouf et al. M.D. Anderson Cancer Center series [8], the need for ureteral reimplantation is not an exclusion criterion for PC at our institution. In this series, we performed 7 unilateral ureteral cases of reimplantation and we were able to achieve SM in 4 patients and the rest had pT0 disease. In terms of the concerning voiding dysfunction after PC, none of the patients in this series underwent any additional procedures for diminished bladder capacity.

Our study is limited by its small size, retrospective nature, surgical selection bias, and relatively short follow-up. Additionally, patients did not receive a uniform NC regimen; 4 (11%) patients had evidence of clinical nodal involvement after NC, and 22 patients (61%) had a variant histology including some with recognized aggressive nature such as small cell (25%) and micropapillary (14%) [14]. Also though none of our patients underwent any additional procedures for voiding dysfunction, we do not have long-term quality of life or medication usage data.

## 5. Conclusion

In this contemporary institutional series, PC after NC in highly selected patients with muscle-invasive bladder cancer provides acceptable oncologic outcomes comparable to those in previously published reports.

## Figures and Tables

**Table 1:** Cohort characteristics.

Number of patients	36
Median age in years (IQR)	69.7 (58.8–76.8)
Median follow-up for entire cohort in months (IQR)	16.8 (9.3–38.7)
Gender	
Male	30 (83%)
Female	6 (17%)
Race	
Caucasian	32 (88.8%)
Other	4 (11.2%)
Urothelial cell carcinoma variant	
None	14 (38.9%)
Small cell	9 (25.0%)
Micropapillary	5 (13.9%)
Other^*^	5 (13.9%)
Not available	3 (8.3%)
Prechemotherapy clinical stage	
cT2	22 (61.1%)
cT3	10 (27.7%)
cT4	2 (5.6%)
Not available	2 (5.6%)
Prechemotherapy carcinoma in situ	
Present	21 (58.3%)
Not present	12 (33.3%)
Not available	3 (8.3%)
Prechemotherapy clinical nodal status	
cN0	28 (77.8%)
cN+	6 (16.7%)
Not available	3 (5.6%)
Chemotherapy regimen	
Gemcitabine and cisplatin	20 (55.5%)
Etoposide and cisplatin	7 (19.4%)
Paclitaxel, ifosfamide, and cisplatin	3 (8.3%)
Methotrexate, vinblastine, doxorubicin, and cisplatin	2 (5.6%)
Other	3 (8.3%)
Not available	1 (2.8%)
Postchemotherapy clinical stage	
cT0	21 (58.3%)
cTis	3 (8.3%)
cT1	1 (2.7%)
cT2	6 (16.7%)
cT3	2 (5.6%)
cT4	1 (2.8%)
Not available	2 (5.6%)
Postchemotherapy CIS	
Present	5 (13.9%)
Not present	26 (72.2%)
Not available	5 (13.9%)
Postchemotherapy clinical nodal status	
cN0	31 (86.1%)
cN+	4 (11.1%)
Not available	1 (2.8%)
Postchemotherapy median follow-up in months (IQR)^∧^	
cT0	17.8 (12.8–34.3)
>cT0	16.8 (4.4–43.1)
Final pathologic stage	
pT0	18 (50.0%)
pTis	6 (16.7%)
pT1	2 (5.6%)
pT2	3 (8.3%)
pT3	7 (19.4%)
Final pathology carcinoma in situ	
Present	14 (38.9%)
Not present	22 (61.1%)
Not available	0 (0%)
Final pathologic nodal involvement	
pN0	27 (75.0%)
pN+	4 (11.1%)
Not available	5 (13.9%)
Surgical margin (SM)	
SM−	15 (41.7%)
SM+	3 (8.3%)
NA (pT0)	18 (50.0%)

^*^Other urothelial cell carcinoma variants include glandular (2) and plasmacytoid (2).

^∧^No difference in median follow-up by rank sum test, *P* = 0.67.

**Table 2:** Clinical restaging after neoadjuvant chemotherapy.

Clinical stage prior to chemotherapy	Number of patients	Clinical stage after chemotherapy							
		cT0	cTa	cTis	cT1	cT2	cT3	cT4	NA
cT2	22	14	0	2	0	5	0	0	1
cT3	10	6	0	1	0	1	2	0	0
cT4	2	0	0	0	1	0	0	1	0
NA	2	1	0	0	0	0	0	0	1

**Table 3:** Pathologic staging after partial cystectomy.

Clinical stage prior to chemotherapy	Number of patients	Pathologic stage after partial cystectomy						
		pT0	pTa	pTis	pT1	pT2	pT3	pT4
cT2	22	13	0	5	0	2	2	0
cT3	10	4	0	0	2	1	3	0
cT4	2	0	0	1	0	0	1	0
NA	2	1	0	0	0	0	1	0

Clinical stage after chemotherapy	Number of patients	pT0	pTa	pTis	pT1	pT2	pT3	pT4

cT0	21	14	0	1	2	2	2	0
cTa	0	0	0	0	0	0	0	0
cTis	3	2	0	1	0	0	0	0
cT1	1	0	0	1	0	0	0	0
cT2	6	1	0	2	0	1	2	0
cT3	2	1	0	0	0	0	1	0
cT4	1	0	0	0	0	0	1	0
NA	2	0	0	1	0	0	1	0

**Table 4:** Sites of first recurrence (*n* = 19).

Site of first recurrence	no.
Bladder only	6
Bladder/retroperitoneum	1
Bladder/pelvis/peritoneum	1
Bladder/lung/liver	1
Pelvis	2
Pelvis/retroperitoneum	1
Pelvis/liver	1
Retroperitoneum	2
Lung	1
Liver	1
Brain	1
Mediastinum	1

**Table 5:** Kaplan-Meier survival estimates for entire cohort.

	Recurrence-free survival	Advanced recurrence-free survival	Overall survival
Median time to IQR in months	23 (5.9–66.2)	66 (7.2–not reached)	79 (20.6–not reached)
2-year (95% CI)	37% (18.6–56.4%)	58% (37.4–74.3%)	71% (48.2–85.5%)
5-year (95% CI)	28% (9.7–50.1%)	51% (28.6–69.5%)	63% (37.6–80.9%)

IQR: interquartile range; CI: confidence interval.

**Table 6:** Univariable analyses for associations with recurrence, advanced recurrence, and overall survival for entire cohort (*n* = 36).

	Recurrence-free survival	Advanced recurrence-free survival	Overall survival
Age^a^	*P* = 0.91	*P* = 0.70	*P* = 0.08
Gender^b^	*P* = 0.54	*P* = 0.85	*P* = 0.65
Before neoadjuvant chemotherapy clinical stage^b^			
>cT2 versus cT2	*P* = 0.03	*P* < 0.01	*P* = 0.17
cN+ versus cN0	*P* = 0.95	*P* = 0.27	*P* = 0.13
cTis versus none	*P* = 0.16	*P* = 0.63	*P* = 0.62
Urothelial variant versus none	*P* = 0.25	*P* = 0.34	*P* = 0.21
After neoadjuvant chemotherapy variables^b^			
cT0 versus >cT0	*P* = 0.33	*P* = 0.49	*P* = 0.53
<cT2 versus ≥cT2	*P* = 0.48	*P* = 0.60	*P* = 0.75
cTis versus none	*P* = 0.10	*P* = 0.29	*P* = 0.04
cN+ versus cN0	*P* < 0.01	*P* < 0.01	*P* < 0.01
After partial cystectomy variables^b^			
pT0 versus >pT0	*P* = 0.12	*P* = 0.50	*P* = 0.99
<pT2 versus ≥pT2	*P* = 0.02	*P* = 0.01	*P* = 0.12
pTis versus none	*P* = 0.70	*P* = 0.50	*P* = 0.75
pN+ versus pN0	*P* = 0.04	*P* = 0.55	*P* = 0.18
SM+ versus SM−	*P* = 0.01	*P* = 0.04	*P* < 0.01

^a^It is performed using univariate Cox regression.

^b^It is performed using log-rank test.
